# Length-Dependent Photoelectric Property of ZnO Nanowires

**DOI:** 10.1186/s11671-022-03715-2

**Published:** 2022-08-18

**Authors:** Ren Ren, Xiaomin Ren, Hao Liu, Yongqing Huang, Weifang Yuan

**Affiliations:** State Key Laboratory of Information Photonics and Optical Communications at BUPT, Zh. I. Alferov Russian-Chinese Joint Laboratory of Information Optoelectronics and Nanoheterostructures, BUPT-HTGD Joint Laboratory of Quantum Optoelectronics and Bivergentum Theory, Beijing University of Posts and Telecommunications, Beijing, 100876 China

**Keywords:** Nanowires, Length-dependent, Oxygen vacancies, Oxygen adsorption capacity, Lifetime

## Abstract

An interesting phenomenon that the photocurrent (the difference between illumination and dark current) of a ZnO nanowire (NW) under a specified voltage increased as its length increased in a certain range was observed previously and it was supposed to be mainly due to a special mean free path effect (MFPE) which caused a special distribution of dark electron density along the length with two higher electron density regions near the two ends of the NW, respectively, and the lower one in the middle part. However, such an explanation would be unreasonable and the true reasons should be the growing-process caused variation of the oxygen adsorption capacity along the NW length and the length-dependent lifetime of photogenerated carriers. Based on this understanding, a theoretical model to properly explain this phenomenon is proposed and the calculation results are in good agreement with the experimental data. This work has introduced an improved insight into the theory of the length-dependent photoelectric property of ZnO NWs.

## Introduction

Photoresponse is one of the most popular methods of researching and characterizing the properties of nanomaterials [1–3]. In 2014, Song et al. observed that the photocurrent of a ZnO NW changed with its length at room temperature [4]. The diameter of the NW is 50 ${\text{nm}}$ and the applied voltage is 10 ${\text{V}}$. It was found that the photocurrent of the NW increased when its length increased from 40 to about 600 ${\text{nm}}$ but decreased as its length increased further. So, there was a peak in the photocurrent-length curve. This is really an interesting phenomenon. To explain this phenomenon, Song et al. assumed that the average dark electron density in a ZnO NW was inversely dependent on its length by reasoning as follows: the electrons moved without collisions in the mean free path; once electrons in valance band were excited into the conduction band at room temperature, they thought the excited electrons only in regions whose length are the mean free path near electrodes could be collected by electrodes without collision; in contrast, those in other region would move with collisions and fall into valance band; the rich electron regions, where the density of electrons was close to that excited by illumination, would be formed near two ends of the NW; the proportion of the high electron density region in the entire ZnO NW decreased with the increasing length, leading to the average electron density of the ZnO NW decreasing and finally saturating. Moreover, the valance band had no more electrons for further light excitation near two ends of the NW. The density of excited electrons contributing to the photocurrent decreased as the length reduces. Therefore, the photocurrent of a ZnO NW was more dependent on the MFPE as its length increased in the range of 40–600 ${\text{nm}}$ and relied strongly on the length as its length increased further. A peak appeared naturally in the photocurrent-length curve. In addition, they made some supplemental works, such as the research that the length-dependent photoinduced electron density from the surface of a ZnO NW which originates from the desorption of electrons confined in the NW surface [5], to improve their theory. It seems that this interesting phenomenon has been explained well.

However, their explanation has some drawbacks. Based on their reasoning, the electrons are distributed evenly in the NW under dark without bias. When the NW connects in the circuit, the excited electrons will be under collision and fall into the valance band as traveling out the mean free path. The middle part of the NW can be considered to consist of many mean free path lengths, and the excited electrons moving state and the number of electrons falling into the valance band should be the same in every mean free path. In other words, the dark electron density distributes evenly in the NW under a bias. It means that the length-dependent average electron density in a ZnO NW they assumed could not be supported by the MFPE. The MFPE should not be taken as a reason that illustrates this phenomenon. At the same time, the shift of the peak photocurrent length under different light intensities had been overlooked. A weakness of their argument is that it could not explain this ignored phenomenon well. And it is not clear the reason that the surface photoinduced electron density depends on the NW length. Consequently, it is necessary to propose a reasonable theory to explain these phenomena.

In this paper, the dependence of the OAC of a ZnO NW on the length has been studied. As its length increases, the OAC of the NW will be enhanced. It can be explained by the growing-process caused variation of oxygen vacancies (OVs) along the NW length. The results show that its depleted region width decreases and its average dark electron density increases, as the NW length decreases. In addition, LPC strongly depends on the NW length. Given the OAC and LPC, a different understanding of the photocurrent change of a ZnO NW is proposed. Based on the analysis of the effect of temperature on OAC, a qualitative analysis to explain the origin of the peak photocurrent length shifting is also given. The calculation results are consistent with the experimental data. This work provides improved insight into the photoreaction of ZnO NWs.

## The Procedure of Model

Because electrical mobility is much larger than hole mobility, we ignore the contribution of holes to the photocurrent [4]. According to Ohm’s Law, we can write the photocurrent of a ZnO NW as expressions (1):

1 $$\Delta I = \frac{US}{L}\Delta \sigma ,\Delta \sigma = \Delta n\mu e$$

where $U$ is the voltage, $L$ is the length of an NW, $S$ is the cross section of an NW, $e$ is the electron charge, $\mu$ is electrical mobility, and $\Delta n$ denotes the difference of electron density between dark and illumination conditions. Ignoring the effect of OAC, $\Delta n$ is $n_{{{\text{pe}}}}$ which is the concentration of photogenerated electrons. Assuming reasonably that electrical mobility is constant in the NW length range we study, the change in the photocurrent of the NW can be reflected reasonably by that of $\Delta n$ as its length varies. At the same time, the Schottky barrier height (SBH) of the NW is inversely dependent on its length, which can be observed clearly in their experiment. The SBH is closely related to the electrical transport of the NW. Generally speaking, the SBH can be modified by three factors: (1) the difference in work function between the semiconductor and contact metal, which is influenced by the level of doping concentration in the semiconductor when keeping the contact metal constant, (2) the surface state of the semiconductor, and (3) the oxide film on the semiconductor surface. However, in this paper, the SBH is only affected by the first factor because the latter two factors are unchanged for the same growing conditions. The high level of average electron density in ZnO NWs will cause low SBH [6]. As a result, the above-mentioned questions are equivalent to explaining why $\Delta n$ and the average electron density in a ZnO NW depend on its length.

It is widely known that the process of oxygen adsorbing and desorbing on a ZnO NW surface: ${\text{O}}_{{\text{2(g)}}} + {\text{e}}^{ - } \rightleftharpoons {\text{O}}_{{\text{2(ad)}}}^{ - }$ and ${\text{O}}_{{\text{2(g)}}} + 2{\text{e}}^{ - } \rightleftharpoons 2{\text{O}}_{{\text{(ad)}}}^{ - }$ [7–9]. The more detailed process has already been described [10]. In a stable environment, the quantity of adsorbed oxygen molecules dominates the number of electrons captured inside the NW, significantly impacting the NW’s conductivity under dark and illumination. It is necessary to know what dominates the quantity of adsorbed oxygen molecules.

As growing ZnO NWs, varied defects usually appear in NWs, resulting in unintentional doping [11, 12]. Defects such as OVs, as we all know, usually dominate the electronic and chemical properties and adsorption behaviors [13], because OVs could provide many sites for oxygen molecules to adsorb on the surface and seize electrons [14–16]. We can find that the higher density of OVs can lead to more oxygen molecules adsorbing on the surface of a ZnO NW [16–18]. Hence, the OAC can be characterized by the density of OVs. Additionally, it is also reasonable to take the density of adsorbed oxygen to describe the OAC [15, 19] because the adsorbed oxygen molecules occupy the position of OVs in the lattice for ZnO materials [20]. During growing a ZnO NW, with its length increasing, the density of its OVs increases and finally saturates [21]. Kayaci et al. observed that the density of OVs began to increase at a ZnO thickness of approximately 40 nm [20]. In addition, the literature [4] shows that the top shape of ZnO NWs grown by Song et al. is a micro-pyramid whose OAC is weaker than that of flake and column [19]. The proportion of the micro-pyramid-shaped surface in the total surface area of the NW increases as its length decreases, which will result in the weaker OAC of the NW. Besides the effects caused by the growing process, OAC can also be affected by temperature and other external factors [22]. Sanghwa observed the oxygen re-adsorption process on a ZnO NW surface under UV illumination in the air because of the Joule heating effect [23]. It shows that a suitable high temperature will enhance the OAC of a ZnO NW. To analyze conveniently, we first do not consider the effects of Joule heating and other factors due to the low Joule power and constant environmental conditions in Ref. [4]. So, given the length-dependent OAC, it is easy to recognize that the adsorbed oxygen density $N_{s}$ on a ZnO NW surface increases when its length increases, leading to an increase in the density of captured electrons which can be proved by the scanning results of surface potential in the report [5].

Hence, we have the length-dependent density of electrons adsorbed $n_{c}$ on the surface of a ZnO NW as Eq. (2) [24]

2 $$n_{c} = \alpha N_{s}.$$

Here, $\alpha$ is the charge transfer coefficient denoting the number of electrons captured by one chemisorbed oxygen molecule. The expression $n_{c}$ means the number of electrostatic carriers confined on a ZnO NW surface, which can be characterized by the surface potential $V_{sp}$. Then, we can derivate the depleted width $r$ by the following expressions (3)–(5) [25].

3 $$V_{sp} = \frac{{2\pi (e\alpha N_{s} )^{2} }}{{\varepsilon N_{d} }},$$

4 $$\lambda_{D} = \left( {\frac{\varepsilon kT}{{2\pi e^{2} N_{d} }}} \right)^{{{\raise0.7ex\hbox{$1$} \!\mathord{\left/ {\vphantom {1 2}}\right.\kern-\nulldelimiterspace} \!\lower0.7ex\hbox{$2$}}}},$$

5 $$r = \lambda_{D} \left( {\frac{{eV_{sp} }}{kT}} \right)^{{{\raise0.7ex\hbox{$1$} \!\mathord{\left/ {\vphantom {1 2}}\right.\kern-\nulldelimiterspace} \!\lower0.7ex\hbox{$2$}}}}.$$

Here, $\varepsilon$ is the dielectric constant, $N_{d}$ is the concentration of donor impurity, $k$ is the Boltzmann constant, $T$ is the absolute temperature. To calculate the average dark electron density $n_{{{\text{dark}}}}$ in a ZnO NW, we should know the average adsorbed electron density of a ZnO NW exposed to air in the dark which can be described by

6 $$n_{ac} = \frac{{2\int_{0}^{L} {\alpha N_{s} dL} }}{RL}.$$

Therefore, $$n_{{{\text{dark}}}}$$ becomes

7 $$n_{{{\text{dark}}}} = n_{0} - n_{{{\text{ac}}}}$$

where $n_{0}$ is the electron density of the NW before it is exposed to air, and $R$ is the radius of the NW. When a ZnO NW length is close to 40 nm, $N_{s}$ will become zero and $n_{{{\text{dark}}}}$ will approximately equal a constant of $n_{0}$. In contrast, as its length is long enough, $N_{s}$ becomes a constant value of $N_{s0}$ and $n_{{{\text{dark}}}}$ will be approximately reduced to Eq. (8) [26]

8 $$n_{{{\text{dark}}}} = n_{0} - \frac{{2\alpha N_{s0} }}{R}.$$

**Fig. 1:**
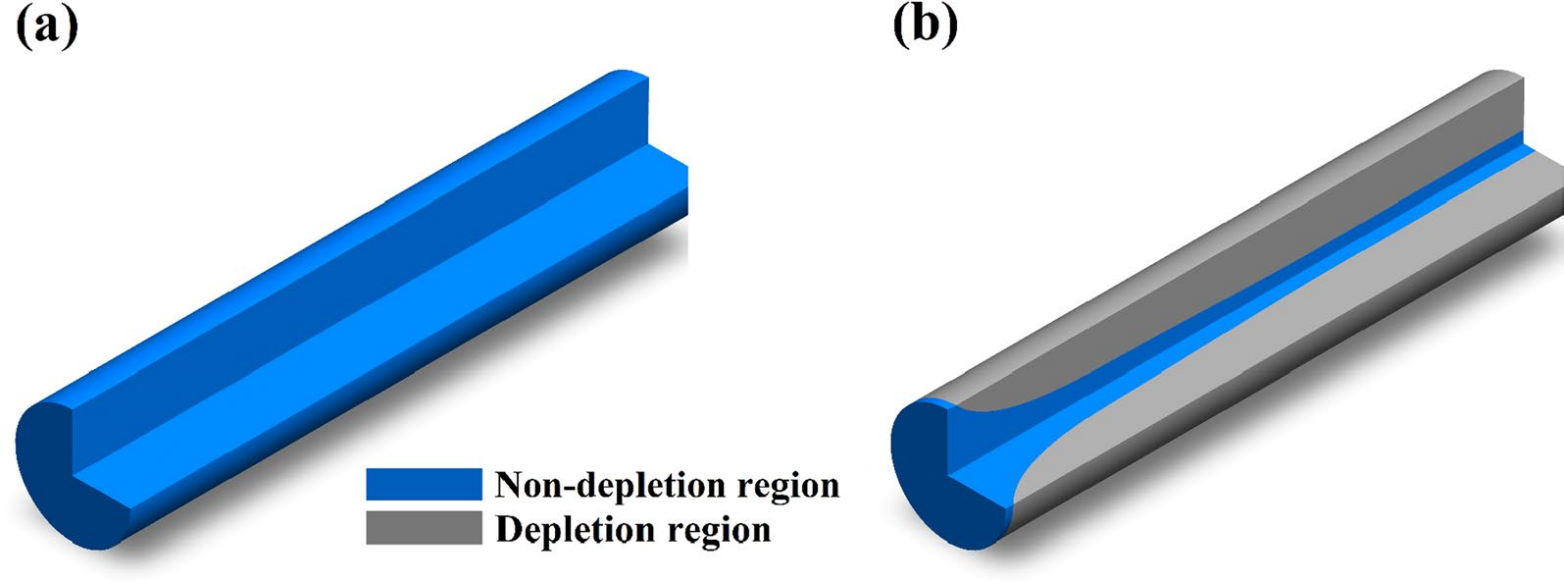
The distribution of electrons of a ZnO NW in vacuum **a** and in the air **b**

As shown in Fig. 1, the distribution of electrons inside a ZnO NW exposed in the air depends strongly on its length, which is different from that in vacuum, and the depleted region width $r$ increases with its length increases and saturates at a long length. When the light which can generate electron-hole pairs illuminates ZnO NWs, the chemisorbed oxygen will be photon-desorbed. The additional photoinduced electron density is determined by the concentration of the electrons confined inside the NW and the light intensity. As the light intensity is large enough, all electrons captured by oxygen molecules on the surface will be desorbed. Thus, $\Delta n$ can be modified as the following expression:

9 $$\Delta n = n_{{{\text{pe}}}} + n_{{{\text{ac}}}}.$$

**Fig. 2:**
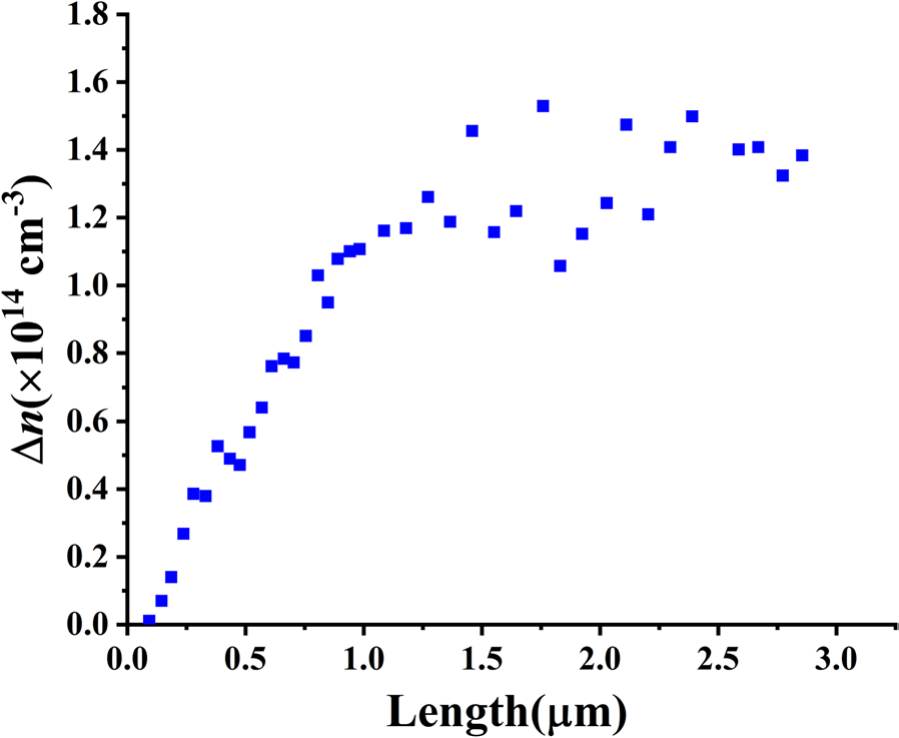
The difference in electron density $\Delta n$ between illumination and dark conditions from the experimental data in Ref. [4]

On the contrary, the number of photon-desorbed electrons is approximately equal to that of the photogenerated holes under low light intensity. The dark electron density in ZnO NWs is about $10^{17} {\text{cm}}^{ - 3}$[4] which is much larger than the electron density ($10^{14} {\text{cm}}^{ - 3}$) that light induced based on our calculation results, as shown in Fig. 2. It reveals that there are enough adsorbed electrons to be photon-desorbed for the NW in Ref. [4]. Therefore, we reasonably believe that $\Delta n$ in Ref. [4] can be instead approximately by $2n_{{{\text{pe}}}}$.

**Fig. 3:**
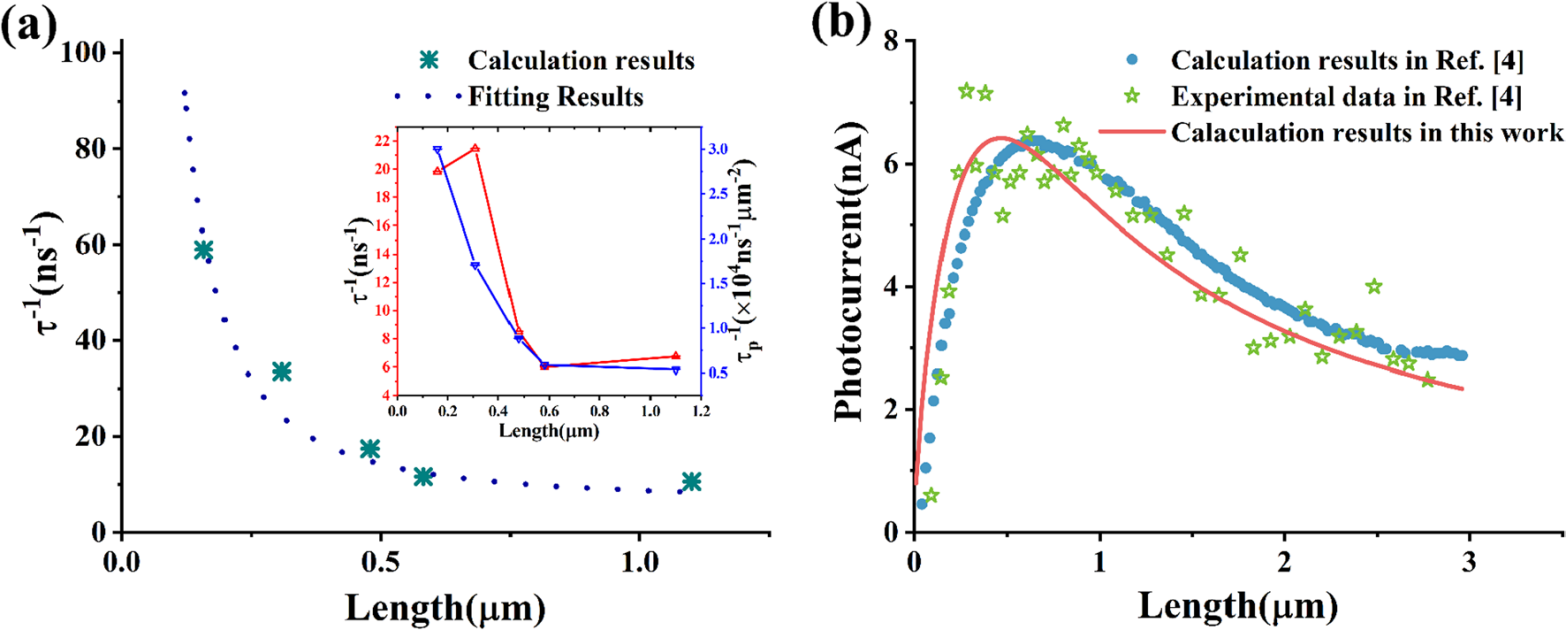
**a** The length-dependent recombination rate of ZnO NWs with a diameter of 50 ${\text{nm}}$ we obtain from the model. Inset: the recombination rate $\tau^{ - 1}$ and the recombination rate per unit area $\tau_{p}^{ - 1}$ are calculated from the experimental data in Ref. [32]. **b** Comparison of the results calculated in this work with the experimental and calculation ones in Ref. [4]. Under a voltage of 10 V, the photocurrent as the function of the height of ZnO NWs with a diameter of 50 nm

Not only can the length of a ZnO NW affect OAC, but in any case, it likewise influences LPC. For ZnO NWs, the higher aspect ratio will result in fewer recombination centers with a small number of interparticle junctions and higher electron delocalization, allowing the electron-hole pairs to separate effectively and lower their recombination rate [15, 17]. The smaller the size, the higher the recombination rate of electron-hole pairs in ZnO NWs [1]. In addition, the decreasing length will cause an increase in electron concentration under dark [6], leading to a decrease in LPC [27]. It should also be noted that OVs can render defect energy levels in a ZnO NW to prevent electron-hole pairs from recombining and lead to stronger light absorption [17, 28–31]. Briefly, the high density of OVs contributes to reducing the recombination rate of electron-hole pairs and increasing the density of photoinduced electrons. More important is that Hong et al. found that the radiative recombination rate in ZnO NWs decreased as the length increased and became saturated at about 600 nm [32]. Amazingly, the length of 600 nm approaches the peak photocurrent length in Ref. [4], which points to the strong relevance between the interesting phenomenon mentioned above and the recombination rate of electron-hole pairs. It is known that LPC $\tau$ is determined by the radiative and nonradiative lifetime: $\frac{1}{\tau } = \frac{1}{{\tau_{r} }} + \frac{1}{{\tau_{nr} }}$ [32]. Here, $\tau_{r}$ and $\tau_{nr}$ are the radiative lifetime and nonradiative lifetime constants, respectively. Considering the effects of the different diameters on the $\tau^{ - 1}$, we obtain the recombination rate per unit area $\tau_{p}^{ - 1}$ from the experimental data of Ref. [32]. The inset in Fig. 3a reveals that the $\tau_{p}^{ - 1}$ is more dependent on the length than the diameter from 29 to 40 nm. It can also be demonstrated in other literature [33]. We believe that $\tau^{ - 1}$ of diameter from 29 to 50 nm in the unit area is similar at the same length. Herein, $\tau^{ - 1}$ of a ZnO NW with a diameter of 50 nm can be calculated and described simply by

10 $$\uptau ^{ - 1} = 2.5 \times L^{ - 1.68} + 6.16,$$

as shown in Fig. 3a. Because test time is far more than $\tau$ which is often less than 1 $ns$ for ZnO materials [34], the density of photogenerated carriers at steady-state follows as

11 $$n_{{{\text{pe}}}} = \beta\upgamma \frac{I}{{{\raise0.5ex\hbox{$\scriptstyle {{\text{hc}}}$} \kern-0.1em/\kern-0.15em \lower0.25ex\hbox{$\scriptstyle \lambda $}}}}\uptau.$$

Here, $\beta$ is quantum efficiency, $I$ is the light illumination intensity, $h$ is Planck’s constant, $c$ is the light speed in vacuum, and $\upgamma$ is the material adsorption factor at the light wavelength $\lambda$ [35], In conclusion, the photocurrent of a ZnO NW in Ref. [4] will be given by

12 $$\Delta I = \frac{USe\mu }{L}(2\beta \gamma \frac{I}{{{\raise0.5ex\hbox{$\scriptstyle {hc}$} \kern-0.1em/\kern-0.15em \lower0.25ex\hbox{$\scriptstyle \lambda $}}}}{\uptau )}.$$

## Results and Discussion

Based on the analysis of OAC and LPC, it is clear that the absorption of ZnO NWs is much more complex under the broadband light source due to OVs and other factors. Thus, to calculate conveniently, we choose $d \times 10^{14} \times \tau$ to fit the experimental data in Ref. [4] after determining the function between $\tau$ and the NW length. $U$, $R$ and $\mu$ are the same as those in Ref. [4]. The calculation results show that $d$ equals 7.22, and the experimental data are found to be in good agreement with our model, as shown in Fig. 3b. The photocurrent of a ZnO NW changes significantly as its length reduces. The photocurrent will decrease slowly rather than stabilize as the length increases more than 3 ${\mu m}$, which seems closer to our model. Maybe additional experimental data will make a more noticeable difference between the theory of us and Song et al. It should also be noted that their measured current is too small to make a high temperature, and electrons in the valence band are challenging to be excited into the conduction band at room temperature because of the wide band gap of ZnO materials (3.4 eV) [36]. So, the electron density they assume near two ends of the NW at room temperature could not be approximately comparable to that excited by illumination. It is not reasonable to use high temperature to explain this interesting phenomenon in Ref. [4]. At the same time, their theory can be found that the high electron density near two ends of the NW remains constant. This cannot illustrate that SBH depends on the NW length.

Furthermore, it is worth noting that the peak photocurrent length in Ref. [5] under increasing light intensities shifts to a longer length. Regrettably, this phenomenon has not been mentioned and explained. It is known that OAC plays an essential role in the photoresponse of ZnO NWs [29, 36, 37]. It can be affected strongly by temperature [23, 38]. Based on the previous reports [38, 39], OAC will be enhanced before reaching the optimal temperature for ZnO nanomaterials. In the air, under illumination, the oxygen molecules re-adsorb on the ZnO NW surface with the increasing temperature caused by Joule heating, significantly affecting the current of the ZnO NW [23]. The increasing illumination intensity makes an increase in the illumination current of the NW, resulting in the temperature caused by Joule heating increasing. In other words, the length corresponding to reaching the same temperature under the Joule heating effect for ZnO NWs becomes longer as the illumination intensity increases. It can be explained simply by $P_{{{\text{Joule}}}} = {{U^{2} S\sigma_{{{\text{light}}}} } \mathord{\left/ {\vphantom {{U^{2} S\sigma_{{{\text{light}}}} } L}} \right. \kern-\nulldelimiterspace} L}$, where $P_{{{\text{Joule}}}}$ is the Joule power and $\sigma_{{{\text{light}}}}$ is the conductivity of ZnO nanomaterials under illumination. To keep $P_{{{\text{Joule}}}}$ constant, the length will be longer when $\sigma_{{{\text{light}}}}$ increases. According to the analysis above, it is clear that the re-adsorption process will significantly accelerate the decrease of $\Delta n$ under the significant Joule heating. Besides, the high temperature makes more electrons excited from the valence band to the conduction band in ZnO NWs under dark conditions to increase the dark current. As a result, the peak photocurrent length of a ZnO NW will shift to a longer length with the increasing illumination light intensity. Combined with the temperature, our model can explain the peak photocurrent length shifting qualitatively under different illumination intensities. However, the theory of Song et al. could not illustrate this interesting phenomenon. According to our theory, the reason why the photoinduced electron density originated from the NW surface and $\Delta n$ change with its length becomes clear. Given the present experimental conditions, it should be mentioned that we have just provided a calculating method and have not given a definite distribution of $N_{s}$ along the growth length of a ZnO NW. A further study with more focus on $N_{s}$ is therefore suggested. Our work provides new insight into the photoresponse of ZnO nanomaterials and may be helpful to optimize devices.

## Conclusions

A length-dependent model to interpret reasonably the photocurrent change of ZnO NWs is proposed based on the OAC and LPC. This study has shown that the length-dependent OAC is due to the shape of ZnO NWs and the growing-process caused variation of OVs along the NW length. Our theory shows that the dark electron density and the width of the depleted region vary when its length changes. Combined with the effect of temperature on OAC, the peak photocurrent length shifting can be explained qualitatively. The calculation results are in good agreement with the experimental data. Our work provides a new understanding of the photoresponse of ZnO NWs.

## Data Availability

The data supporting the findings of this study are available from the corresponding author on reasonable request.

## Abbreviations

NW: Nanowire
MFPE: Mean free path effect
OAC: Oxygen adsorption capacity
LPC: Lifetime of photogenerated carriers
OVs: Oxygen vacancies
SBH: Schottky barrier height
